# The right inferior frontal gyrus as pivotal node and effective regulator of the basal ganglia-thalamocortical response inhibition circuit

**DOI:** 10.1093/psyrad/kkad016

**Published:** 2023-10-13

**Authors:** Qian Zhuang, Lei Qiao, Lei Xu, Shuxia Yao, Shuaiyu Chen, Xiaoxiao Zheng, Jialin Li, Meina Fu, Keshuang Li, Deniz Vatansever, Stefania Ferraro, Keith M Kendrick, Benjamin Becker

**Affiliations:** The Center of Psychosomatic Medicine, Sichuan Provincial Center for Mental Health, Sichuan Provincial People's Hospital, The University of Electronic Science and Technology of China, Chengdu, Sichuan Province 611731, China; Center for Cognition and Brain Disorders, The Affiliated Hospital of Hangzhou Normal University, Hangzhou, Zhejiang Province 311121, China; School of Psychology, Shenzhen University, Shenzhen 518060, China; The Center of Psychosomatic Medicine, Sichuan Provincial Center for Mental Health, Sichuan Provincial People's Hospital, The University of Electronic Science and Technology of China, Chengdu, Sichuan Province 611731, China; Institute of Brain and Psychological Sciences, Sichuan Normal University, Chengdu, 610068, China; The Center of Psychosomatic Medicine, Sichuan Provincial Center for Mental Health, Sichuan Provincial People's Hospital, The University of Electronic Science and Technology of China, Chengdu, Sichuan Province 611731, China; Center for Cognition and Brain Disorders, The Affiliated Hospital of Hangzhou Normal University, Hangzhou, Zhejiang Province 311121, China; The Center of Psychosomatic Medicine, Sichuan Provincial Center for Mental Health, Sichuan Provincial People's Hospital, The University of Electronic Science and Technology of China, Chengdu, Sichuan Province 611731, China; Brain Cognition and Brain Disease Institute (BCBDI), Shenzhen Institute of Advanced Technology, Chinese Academy of Sciences, Shenzhen 518055, China; The Center of Psychosomatic Medicine, Sichuan Provincial Center for Mental Health, Sichuan Provincial People's Hospital, The University of Electronic Science and Technology of China, Chengdu, Sichuan Province 611731, China; The Center of Psychosomatic Medicine, Sichuan Provincial Center for Mental Health, Sichuan Provincial People's Hospital, The University of Electronic Science and Technology of China, Chengdu, Sichuan Province 611731, China; The Center of Psychosomatic Medicine, Sichuan Provincial Center for Mental Health, Sichuan Provincial People's Hospital, The University of Electronic Science and Technology of China, Chengdu, Sichuan Province 611731, China; School of Psychology and Cognitive Science, East China Normal University, Shanghai 200062, China; Institute of Science and Technology for Brain-Inspired Intelligence, Fudan University, Shanghai 200433, China; The Center of Psychosomatic Medicine, Sichuan Provincial Center for Mental Health, Sichuan Provincial People's Hospital, The University of Electronic Science and Technology of China, Chengdu, Sichuan Province 611731, China; The Center of Psychosomatic Medicine, Sichuan Provincial Center for Mental Health, Sichuan Provincial People's Hospital, The University of Electronic Science and Technology of China, Chengdu, Sichuan Province 611731, China; Institute of Science and Technology for Brain-Inspired Intelligence, Fudan University, Shanghai 200433, China; State Key Laboratory of Brain and Cognitive Sciences, The University of Hong Kong, Hong Kong 999077, China; Department of Psychology, The University of Hong Kong, Hong Kong 999077, China

**Keywords:** response inhibition, basal ganglia, inferior frontal gyrus, effective connectivity, DCM

## Abstract

**Background:**

The involvement of specific basal ganglia-thalamocortical circuits in response inhibition has been extensively mapped in animal models. However, the pivotal nodes and directed causal regulation within this inhibitory circuit in humans remains controversial.

**Objective:**

The main aim of the present study was to determine the causal information flow and critical nodes in the basal ganglia-thalamocortical inhibitory circuits and also to examine whether these are modulated by biological factors (i.e. sex) and behavioral performance.

**Methods:**

Here, we capitalize on the recent progress in robust and biologically plausible directed causal modeling (DCM-PEB) and a large response inhibition dataset (*n* = 250) acquired with concomitant functional magnetic resonance imaging to determine key nodes, their causal regulation and modulation via biological variables (sex) and inhibitory performance in the inhibitory circuit encompassing the right inferior frontal gyrus (rIFG), caudate nucleus (rCau), globus pallidum (rGP), and thalamus (rThal).

**Results:**

The entire neural circuit exhibited high intrinsic connectivity and response inhibition critically increased causal projections from the rIFG to both rCau and rThal. Direct comparison further demonstrated that response inhibition induced an increasing rIFG inflow and increased the causal regulation of this region over the rCau and rThal. In addition, sex and performance influenced the functional architecture of the regulatory circuits such that women displayed increased rThal self-inhibition and decreased rThal to GP modulation, while better inhibitory performance was associated with stronger rThal to rIFG communication. Furthermore, control analyses did not reveal a similar key communication in a left lateralized model.

**Conclusions:**

Together, these findings indicate a pivotal role of the rIFG as input and causal regulator of subcortical response inhibition nodes.

## Introduction

Animal models and human neuroimaging studies convergently demonstrated that inhibitory control critically relies on highly specific basal ganglia-thalamocortical circuits (Alexander *et al*., 1986, 1991; Alexander and Crutcher, 1990; Aron *et al*., 2007; Jahfari *et al*., 2019; Morein-Zamir and Robbins, 2015; Pfeifer *et al*., 2022; Schall and Godlove, 2012; Stuphorn, 2015; Verbruggen and Logan, 2009; Wei and Wang, 2016). Dysregulations in this circuit have been implicated in disorders characterized by inhibitory control deficits, including addiction (Klugah‐Brown *et al*., 2020; Morein-Zamir and Robbins, 2015; Zhou *et al*., 2018), attention deficit/hyperactivity (ADHD, Morein‐Zamir *et al*., 2014; Sonuga-Barke, 2005), schizophrenia (Camchong *et al*., 2006; Feng *et al*., 2018; Mamah *et al*., 2007), and Parkinson disorder (DeLong and Wichmann, 2015; Obeso *et al*., 2000).

The key nodes within this response inhibition circuitry have been extensively mapped with convergent evidence suggesting critical contributions from the pre-supplementary motor area (pre-SMA) and lateral prefrontal cortex (lPFC), in particular, the inferior frontal gyrus (IFG) (Aron *et al*., 2003; Dambacher *et al*., 2014; Hampshire *et al*., 2010; Maizey *et al*., 2020; Schaum *et al*., 2021; Verbruggen and Logan, 2008; Zhang *et al*., 2017) and the striatal regions including the caudate and putamen (Eagle *et al*., 2011; Ghahremani *et al*., 2012; Hampton *et al*., 2017; Kelly *et al*., 2004; Ott and Nieder, 2019; Robertson *et al*., 2015; Robbins, 2007). Importantly, consistent evidence has demonstrated that enhanced behavioral response inhibition was closely associated with increased connectivity strength in the IFG--striatal pathway (Jahfari *et al*., 2011; Xu *et al*., 2016). Furthermore, anatomical and neurochemical studies suggest that response inhibitory control within the fronto-striatal circuitry is modulated by dopaminergic and noradrenergic signaling (Bari *et al*., 2011; Ghahremani *et al*., 2012; Li *et al*., 2020; Pfeifer *et al*., 2022; Rae *et al*., 2016; Robertson *et al*., 2015). Such as, dopamine receptor availability in the fronto-striatal circuits is significantly related to inhibition-related neural responses (Ghahremani *et al*., 2012; Pfeifer *et al*., 2022). While the dorsal striatum represents an important locus of dopaminergic control of response inhibition (Ghahremani *et al*., 2012; Robertson *et al*., 2015), dopamine receptor availability in the lPFC modulates motor control via downstream regulatory projections to the striatum (Ott and Nieder, 2019; Vijayraghavan *et al*., 2016). On the other hand, enhanced norepinephrine signaling facilitates response inhibition via modulation of the IFG and its connections with the striatum (Chamberlain *et al*., 2009; Rae *et al*., 2016), during which the IFG plays an important role in top-down control of the basal ganglia regions (Buschman and Miller, 2014; Hampshire *et al*., 2010; Jahfari *et al*., 2012; Kim, 2014; Puiu *et al*., 2020; Renteria *et al*., 2018; Schaum *et al*., 2021; Tops and Boksem, 2011).

A large number of studies have demonstrated the pivotal role of the thalamus in the basal ganglia-thalamocortical model of response inhibition (Alexander *et al*., 1986, 1991; Alexander and Crutcher, 1990). Specifically, the thalamus relays information between the basal ganglia and cortex (Collins *et al*., 2018; Haber and Mcfarland, 2001; Haber and Calzavara, 2009; McFarland and Haber, 2002)—thus facilitating response inhibition and performance monitoring (Bosch-Bouju *et al*., 2013; Huang *et al*., 2018; Saalmann and Kastner, 2015; Tanaka and Kunimatsu, 2011)—via dense reciprocal connections with the basal ganglia and PFC (Guillery, 1995; Phillips *et al*., 2021; Xiao *et al*., 2009; Tanaka and Kunimatsu, 2011).

Consistent findings from animal model and human neuroimaging studies show that the globus pallidus (GP) also plays an essential role in action execution and response inhibition (Casey *et al*., 1997; Mallet *et al*., 2016; Pan *et al*., 2018; Wei and Wang, 2016). A previous structural imaging study revealed that a better behavioral performance during a response inhibition task was related to a larger GP volume (Casey *et al*., 1997). In addition, a study from Wei and Wang showed that GABAergic inhibitory projections from the external segment of the GP to the striatum are crucial for inhibiting a planned response (Wei and Wang, 2016).

Several studies have explored sex differences in response inhibition performance and the associated neural activity (Chung *et al*., 2020; Ribeiro *et al*., 2021; Rubia *et al*., 2013; Sjoberg and Cole, 2018). While the existing evidence from most studies and meta-analyses showed no significant sex difference on behavioral performance (Chung *et al*., 2020; Cross *et al*., 2011; Gaillard *et al*., 2021; Garavan *et al*., 2006; Li *et al*., 2006), some other studies showed that female individuals demonstrate higher accuracy and faster stop signal reaction times compared to male participants (Ribeiro *et al*., 2021; Rubia *et al*., 2013; Sjoberg and Cole, 2018) and one study reported that males demonstrate better response inhibition compared to females (Gaillard *et al*., 2020). With respect to neural differences the previous literature remained inconsistent and the direction of sex differences may additionally vary depending on the task administered (Go/NoGo task or stop signal task) and the age of the participants (Chung *et al*., 2020; Rubia *et al*., 2013; Weafer, 2020). Such as, some studies reported that male participants tend to display greater brain activity in frontal as well as motor control-related regions such as the GP and thalamus during response inhibition on stop signal tasks when inhibiting an already-initiated response (Li *et al*., 2006, 2009), while female participants tend to display greater brain activity during inhibition on Go/NoGo tasks when inhibiting the initiation of a response (Chung *et al*., 2020; Garavan *et al*., 2006).

Convergent evidence from human lesion studies and neuroimaging meta-analyses demonstrates a right-lateralized inhibitory control network encompassing the right IFG (rIFG), right caudate nucleus (rCau), right GP (rGP), and right thalamus (rThal) (Aron *et al*., 2003; Chevrier *et al*., 2007; Garavan *et al*., 1999; Hung *et al*., 2018; Jahfari *et al*., 2011; Thompson *et al*., 2021). However, while extensive research has highlighted the critical role of these regions within a right-lateralized inhibitory control circuitry, the causal information flow and critical contribution of single nodes within this network as well as the modulatory effect of sex have not been determined.

We therefore capitalized on a novel dynamic causal modeling (DCM) approach based on *a priori* specification of biologically and anatomically plausible models that allows estimation of directed causal influences between nodes and their modulation by changing task demands (Friston *et al*., 2003; Stephan *et al*., 2010) in the largest sample to date (*n* = 250). The DCM approach conceptualizes the brain as a nonlinear dynamical input-state-output system and was developed to provide a more biologically informed approach to test a hypothesis about experimental manipulation-dependent interactions between brain regions based on differential equations describing interactions between neural populations that may directly or indirectly give rise to the observed functional magnetic resonance imaging (fMRI) data. The estimated parameters in these models are considered as directed or effective connectivity between brain regions. DCM further allows comparison of modulatory effective connectivity strength across different experimental conditions using Bayesian contrasts (Dijkstra *et al*., 2017) and, in combination with the recently developed parametrical empirical Bayes (PEB) hierarchical framework (DCM-PEB method), it allows modeling of both commonalities and differences in effective connectivity between participants, e.g. to determine the neurobiological basis of sex and behavioral performance variations (Friston *et al*., 2016; Zeidman *et al*., 2019a, 2019b).

To determine the causal information flow and critical nodes in the basal ganglia-thalamocortical circuits and whether these are modulated by biological factors (i.e. sex) and show functional relevance in terms of associations with performance we capitalized on DCM-PEB in combination with fMRI data collected in a large sample of healthy individuals (*n* = 250) during a well-established response inhibition paradigm (emotional Go/NoGo task, see also Zhuang *et al*., 2021). To unravel the key nodes and causal influences within the inhibitory control network, we first estimated the effective connectivity between and within key regions involved in response inhibitory control within the rIFG-rCau-rGP-rThal functional circuit (right lateralized model) and, second, we estimated sex differences and behavioral performance effects on connectivity parameters. Furthermore, to validate the hemispheric asymmetry of the inhibitory control network, an identical model of nodes was tested in the left hemisphere (left lateralized model).

Given convergent evidence on a pivotal role of the right IFG in mediating top-down cortical--subcortical control via connectivity pathways with striatal and thalamic areas during response inhibition (Aron *et al*., 2003; Dambacher *et al*., 2014; Hampshire *et al*., 2010; Maizey *et al*., 2020), we predicted a greater modulatory effect on rIFG and its directed connectivity to both rCau and rThal in the NoGo compared to Go condition. Additionally, based on previous studies reporting sex differences in both, behavioral response inhibition and associated neural processing in cortical-subcortical circuits (i.e. sex, Li *et al*., 2006; Ribeiro *et al*., 2021; Sjoberg and Cole, 2018), as well as a significant correlation between enhanced inhibitory control and increased frontal-striatal connectivity (Chang *et al*., 2020; Jahfari *et al*., 2011; Wei and Wang, 2016; Xu *et al*., 2016), we hypothesized a modulation of the key pathways by biological and performance variations with better response inhibition being associated with stronger causal regulation in the inhibition circuitry, especially in the IFG-Cau pathway. Finally, in line with consistent evidence that showed right-lateralized brain areas and neural circuits involved in the response inhibition (Aron *et al*., 2003; Chevrier *et al*., 2007; Hung *et al*., 2018; Jahfari *et al*., 2011; Thompson *et al*., 2021), we proposed a different causal structure for the left and right models given the hemispheric asymmetry in the inhibitory network.

## Materials and Methods

### Participants

In this study, *n* = 250 healthy right-handed participants were enrolled and underwent a validated Go/NoGo fMRI paradigm. The data have been previously used to examine undirected functional connectivity within domain-general and emotion-specific inhibitory brain systems (Zhuang *et al*., 2021), and were part of a larger neuroimaging project examining pain empathy (Li *et al*., 2019 ; Zhou *et al*., 2020), emotional face memory (Liu *et al*., 2022), and mirror neuron processing (Xu *et al*., 2022). After quality assessment during the processes of data collection and preprocessing *n* = 218 participants were included (104 males, details see Supplementary Materials). During the model estimation processes, explained variance by the specified model on the individual level was calculated with higher values reflecting better model inversion (Zeidman *et al*., 2019a). In line with previous studies (Bencivenga *et al*., 2021; Rupprechter *et al*., 2020), participants with <10% of explained variance were excluded and finally a total of 118 participants (56 males, age: mean ± SEM = 21.57 ± 0.21 years) were included into further analyses. The study was approved by the local ethics committee and in accordance with the latest version of the Declaration of Helsinki.

### Response Inhibition Paradigm

A validated mixed event-related block design linguistic emotional Go/NoGo fMRI paradigm was employed (Goldstein *et al*., 2007; Protopopescu *et al*., 2005, for details see Zhuang *et al*., 2021). Notably, although both the Go/NoGo and stop signal paradigm are commonly used to examine response inhibition control and associated brain function, the former paradigm captures action restraint while the latter primarily involves action cancellation (Raud *et al*., 2020; Schachar *et al*., 2007). During the present Go/NoGo task, participants were required to make responses as accurately and quickly as possible based on orthographical cues, i.e. words were presented in normal or italic font. For words in a normal font, participants were instructed to perform a button-press (Go trials), while inhibiting their response to words presented in italic font (NoGo trials). Omission errors were defined when no responses were made for Go trials, while commission errors were defined when responses were made to NoGo trials. Positive, negative, and neutral words were included into the paradigm as stimuli. However, given that the main aim of the present study was to examine the causal influence within the general inhibition network as proposed by Alexander *et al*. (1986, 1991; Alexander and Crutcher, 1990) and to increase statistical power in this respect the different emotional valence conditions (e.g. positive Go condition, positive NoGo condition, negative Go condition, negative NoGo condition, neutral Go condition, and neutral NoGo condition) were not further accounted for in the DCM analysis. Stimuli were presented in two runs and each run included 12 blocks (six blocks: Go; six blocks: NoGo). Each Go block encompassed 18 normal font words (100% Go trials) while each NoGo block encompassed 12 normal font words (66.7% Go trials) and six italicized font words (33.3% NoGo trials). Further details can be found in Zhuang *et al*. (2021) and the Supplementary Materials.

### Behavioral Data Analysis

In our previous study, we demonstrated that participants exhibited more commission errors during inhibitory control (i.e. NoGo > Go) as well as faster responses in positive Go contexts and lower accuracy in positive NoGo contexts (Zhuang *et al*., 2021). Given that sex-differences were examined in the DCM model, the present analyses additionally examined sex-differences on accuracy and reaction times (Supplementary Materials). Given previous studies have showed age-related effects on inhibition (Rey-Mermet *et al*., 2018; Rubia *et al*., 2007) age was included as covariate.

### MRI Data Acquisition and Preprocessing

MRI data were collected on a 3T MRI system using standard sequences and were initially preprocessed using validated protocols in SPM 12 (for details see Supplementary Materials).

### GLM Analysis

An event-related general linear model (GLM) was established in SPM12. To examine domain general inhibitory control (irrespective of emotional context) the overarching inhibitory control contrast was modeled (e.g. all NoGo > all Go trials) and convolved with the canonical hemodynamic response function. Six head motion parameters were included in the design matrix to control movement-related artifacts and a high-pass filter (1/128 Hz) was applied to remove low frequency components. The contrast of interest (contrast: NoGo > Go) was created and subjected to one-sample *t*-test at the second level. In line with previous studies (Aron *et al*., 2003; Chevrier *et al*., 2007; Hung *et al*., 2018; Jahfari *et al*., 2011; Thompson *et al*., 2021), group-level (contrast: NoGo > Go) peaks in the IFG, Cau, GP, and Thal within the identified general inhibition network were then used to define individual-specific regions of interest (ROI) for the DCM analysis. Additionally, a two-sample *t*-test was conducted (contrast: NoGo > Go) to examine sex-dependent effects on the response inhibition network. Analyses were corrected for multiple comparisons using a conservative peak-level threshold on the whole brain level (*P* < 0.05 family-wise error, FWE).

### DCM and Node Definition

A DCM analysis was employed to determine directed causal influences according to the circuitry model proposed by Alexander *et al*. (1986, 1991; Alexander and Crutcher, 1990). The DCM approach allows construction of a realistic neuronal model of interacting regions and the prediction of the underlying neuronal activity from the measured hemodynamic response (Friston *et al*., 2003; Stephan *et al*., 2007). To this end, directed causal influences between the key regions including IFG, Cau, GP, and Thal in the basal ganglia-thalamocortical loop and their modulation via experimental manipulations (engagement of motor inhibitory control) were examined. In line with previous neuroimaging studies and meta-analyses demonstrating a right-lateralized inhibition model (right model) encompassing the rIFG, rCau, rGP, and rThal (Aron *et al*., 2003; Chevrier *et al*., 2007; Hung *et al*., 2018; Jahfari *et al*., 2011; Thompson *et al*., 2021), our main hypothesis testing focused on the right lateralized network. To further validate the hemispheric asymmetry of the inhibitory control network an identical model was tested for the left hemisphere including the lIFG, lCau, lGP, and lThal. In line with previous studies, we combined atlas-based masks (Human Brainnetome Atlas, Fan *et al*., 2016) with group-level and individual level activity maps to generate the corresponding nodes (Fernández-Espejo *et al*., 2015; Holmes *et al*., 2021; Qiao *et al*., 2020; Van Overwalle *et al*., 2020). Among this, the caudate is limited to a mask that combines the ventral and dorsal caudate but not the putamen (Fan *et al*., 2016).

### Model Specification and Estimation

A two-step DCM analysis was performed using the DCM-parametric empirical Bayes (PEB) approach (Zeidman *et al*., 2019a, 2019b). On the first-level, time-series from four ROI (rIFG, rCau, rGP, rThal) were extracted. A full DCM model was specified for each participant and all connectivity parameters in both forward (e.g. rIFG-rThal-rGP-rCau-rIFG) and backward (e.g. rIFG-rCau-rGP-rThal-rIFG) directions were estimated. We estimated three key DCM parameters: (i) the matrix A reflecting all connections including forward and backward connectivity between ROI and self-inhibitions in each ROI, (ii) the matrix B representing modulatory effects of Go and NoGo condition on all connections, and (iii) the matrix C representing the driving inputs into ROI from Go and NoGo conditions separately. Given that all inputs in the model were mean-centered, intrinsic connectivity in the matrix A indicates mean effective connectivity independent of all experimental conditions. The model was estimated using variational Laplace (Friston *et al*., 2007). Further details are presented in the Supplementary Material. At the second (group) level, we constructed a PEB model over the first-level estimated parameters. In accordance with previous studies (Bencivenga *et al*., 2021; Rupprechter *et al*., 2020), we evaluated the explained variance by the model on the individual level (Zeidman *et al*., 2019a)—and then we only included participants with >10% of explained variance in the PEB model. Finally, 118 participants were included for further analyses.The number of excluded participants is similar to a previous study (Rupprechter *et al*., 2020). The differences on behavioral performance were examined between the excluded and included participants and no significant differences were found (all *P* ≥ 0.23, for details see the Supplementary Material), suggesting no evidence of biased selection.

The primary aim of the present study was to establish a causal neurobiological model for response inhibition and to determine the interaction between key players in this circuitry. To evaluate the model three PEB analyses were carried out separately for A, B, and C matrices. Separate analyses examined sex and performance variations (for details, see the Supplementary Materials).

Next, to identify the model that best represented our data, Bayesian model reduction was performed to compare the free energy of the full model with numerous reduced models for which specific parameters were “switched off” (Friston *et al*., 2016). An automatic greedy search procedure (iterative procedure) was employed to facilitate an efficient comparison of thousands of models. In this procedure, parameters that do not contribute to free energy were pruned away. Next, the Bayesian model average, performing a weighted average of the parameters of each model, was calculated over the 256 models obtained from the final iteration (Friston *et al*., 2016).

Finally, to compare the effective connection strength, especially the cortical-subcortical connectivity and driving inputs into each region from different experimental conditions (NoGo and Go conditions), Bayesian contrasts (Dijkstra *et al*., 2017) were computed over parameters from the B and C matrices. Group-level estimated parameters were thresholded at posterior probability >95% (indicating strong evidence: Kass and Raftery, 1995) based on free energy.

## Results

### Behavioral Results

The two-way repeated-measures ANOVA on accuracy found a significant main effect of inhibition [*F*(1115) = 21.73, *P* < 0.001, *η*_p_^2^ = 0.16], with a higher accuracy for Go compared to NoGo trials (Go trials: mean ± SEM = 98.47% ± 0.31, No Go trials: mean ± SEM = 70.34% ±1.44, Cohen's *d* = 2.48). No sex differences were found for accuracy or reaction times (*P* > 0.18). The mean reaction time for correct Go trials is mean ± SEM = 314.44, ms ± 4.94.

### BOLD Activation (GLM) Analysis

**Figure 1:**
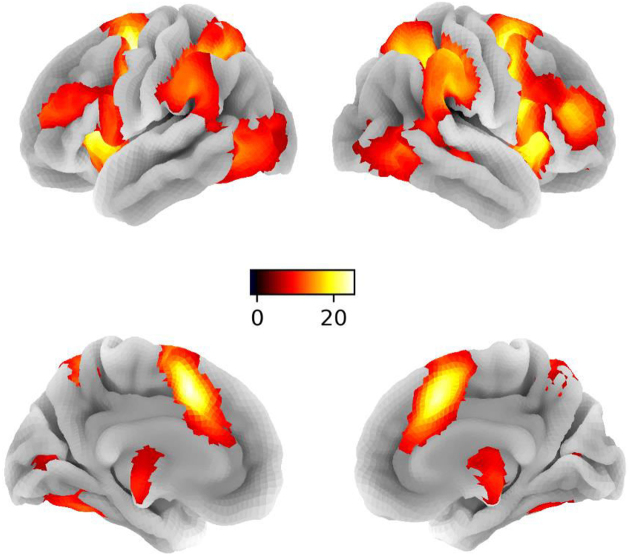
Brain activation maps for general response inhibition on whole brain level (contrast: NoGo > Go; *P* < 0.05 FWE, peak level). L, left; R, right. The color bar represents the *t*-values of the BOLD signal and reflect the significance level of the contrast.

**Figure 2:**
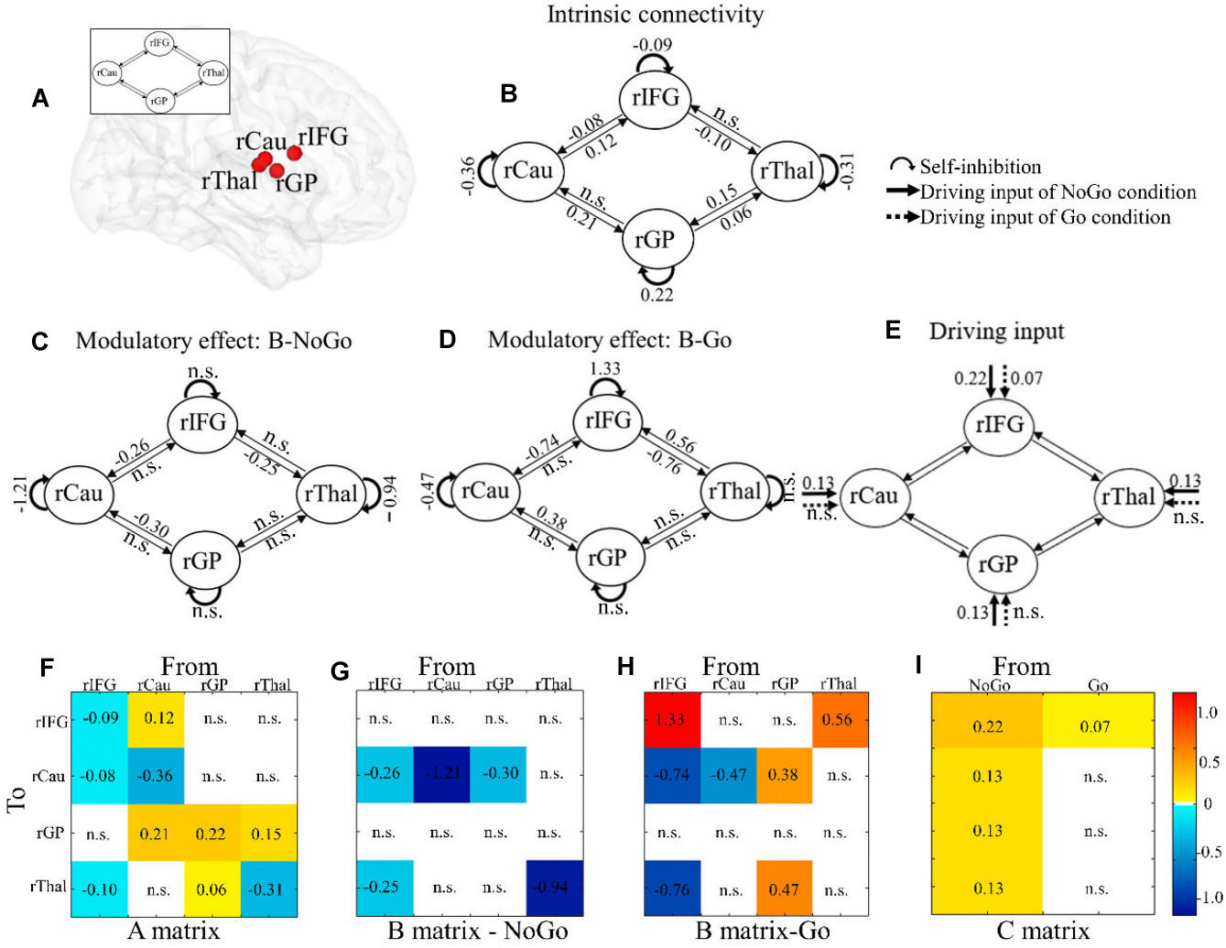
Location of regions included in the right model and group-level connectivity parameters. (**A**) Location of regions included in the right model. The A matrix: intrinsic connectivity across all experimental conditions (**B, F**). The B matrix: modulatory effect on effective connectivity between regions and self-inhibitions from NoGo (**C, G**) and Go condition (**D, H**). The C matrix: Driving inputs in ROI in the NoGo and Go condition (**E**, *I*). Values in matrices reflect the connectivity parameters. Effective connectivity strengths are displayed by the color ranging from yellow to dark red (i.e. excitatory connectivity) and from turquoise to dark blue (i.e. inhibitory). Parameters with stronger evidence (posterior probability >95%) are presented and subthreshold parameters are marked with “n.s.”.

Examination of domain general inhibition (contrast: NoGo > Go) revealed a widespread fronto-parietal cortical and thalamo-striatal subcortical network including the IFG, striatal, pallidal, and thalamic regions (Fig. 1 and Table 1) during response inhibition. Group-level peaks in the rIFG, rCau, rGP, and rThal were selected as centers of the ROI for model testing (Fig. 2a and Table 2). No significant sex difference was observed in blood oxygen level-dependent (BOLD) activation.

**Table 1:** Regions involved in the domain general inhibition control on the group level (contrast: NoGo > Go).

		Coordinates			
Regions	Cluster *k*	*x*	*y*	*z*	*t* value
Frontal lobe including MFG and SFG extending to parietal, temporal and occipital lobe	19 718	27	3	51	25.40
		18	6	57	25.05
		−3	12	45	24.55
mOFC	72	27	45	−21	8.67
lCalcarine	152	−12	−75	9	8.44
rCalcarine	61	15	−72	9	6.57

Note: Group level brain activation maps involved in the domain general inhibition control (contrast: NoGo > Go, peak level, p_FWE_ < 0.05). MFG, middle frontal gyrus; SFG, superior frontal Gyrus; mOFC, middle orbital frontal cortex; r, right; l, left.

**Table 2:** Activation and peak values for key regions included in the right model.

		Coordinates			
Regions	Cluster *K*	*x*	*y*	*z*	*t*-value
rIFG	611	51	12	18	21.40
rCau	144	15	−3	15	13.61
rGP	63	21	−3	9	12.43
rThal	340	15	−6	12	14.30

Note: Key nodes including rIFG, rCau, rGP, and rThal survived from the overlay between image masks of corresponding regions defined by Human Brainnetome Atlas and group level brain activation maps (peak level, p_FWE_ < 0.05) and thus served as ROI combined with the individual peak location search on the individual level. Cau, caudate nucleus; GP, global pallidum; r, right; Thal, thalamus.

### Causal Connectivity (DCM) Analysis

For the matrix A, the diagonal cells represent self-connections that are unitless log scaling parameters and were multiplied with the default value of −0.5 Hz (Zeidman *et al*., 2019a). Positive values indicate increased self-inhibition due to task condition and decreased responsivity to the inputs from the other regions of the network, while negative values indicate decreased self-inhibition and increased responsivity to the inputs from other nodes of the network (Zeidman *et al*., 2019a). Our findings revealed negative self-inhibition values for the rIFG, rCau, and rThal but a positive value for the rGP (Fig. 2b,f), indicating that the GP increased self-connection while the other nodes increased interaction with other nodes in the network.

For the off-diagonal cells in the matrix A, the values (in Hz) reflect the rate of change in the activity of the target region caused by the source region per second. Positive values reflect excitatory effects while negative values indicate inhibitory effects. In the forward direction (e.g. rIFG-rThal-rGP-rCau-rIFG), we found a significant negative connectivity from rIFG to rThal and positive connectivity from rThal to rGP as well as rCau to rIFG. In the backward direction (e.g. rIFG-rCau-rGP-rThal-rIFG), rIFG exhibited a negative inhibitory influence onto rCau, alongside an excitatory connection from rCau to rGP and rGP to rThal (Fig.   2b,f). Although the connectivity from rThal to rIFG was not significant, a weak evidence (posterior probability of 57%) for this connection was observed with a more lenient threshold.

Values in the matrix B represent the rate of change, in Hz, in the connectivity from source area to target area induced by the experimental conditions (Zeidman *et al*., 2019a). During inhibitory control (NoGo condition) the rIFG exerted a negative influence onto the rCau and rThal whereas the rGP exerted a negative influence on the rCau (Fig. 2c,g). In addition, we found negative self-inhibition values in both rCau and rThal, respectively. During the Go condition a negative influence of the rIFG on both rCau and rThal was observed (Fig. 2d,h), while the positive influence was observed from the rGP to rCau and from rThal to rIFG. Moreover, we found a positive self-inhibition value in rIFG and a negative value in rCau. A Bayesian contrast (NoGo > Go) allowed us to compare the connectivity strength modulation during the different experimental conditions and revealed a very strong evidence (posterior probability > 99%) that the causal influence of the rIFG to both, the rCau and rThal was stronger during inhibitory control (NoGo vs Go condition). This reflects that response inhibition critically requires a causal top-down cortical-subcortical regulation via the right IFG. We additionally found a very strong evidence (posterior probability > 99%) for a considerably stronger inhibitory connectivity from rGP to rCau in the NoGo compared to Go condition.

The matrix C represents the rate of change in neural response of one brain region due to the driving input from an experimental condition (Zeidman *et al*., 2019a). During inhibitory control (NoGo) all regions (rIFG, rCau, rGP, and rThal) exhibited excitatory driving input while during the Go condition only the rIFG exhibited excitatory input (Fig. 2e,i). Bayesian contrasts directly comparing the conditions (NoGo > Go) demonstrated an increasing driving input specifically in the rIFG during engagement of cognitive control (NoGo > Go condition) with a 100% posterior probability.

### Sex Differences in Connectivity Parameters

**Figure 3:**
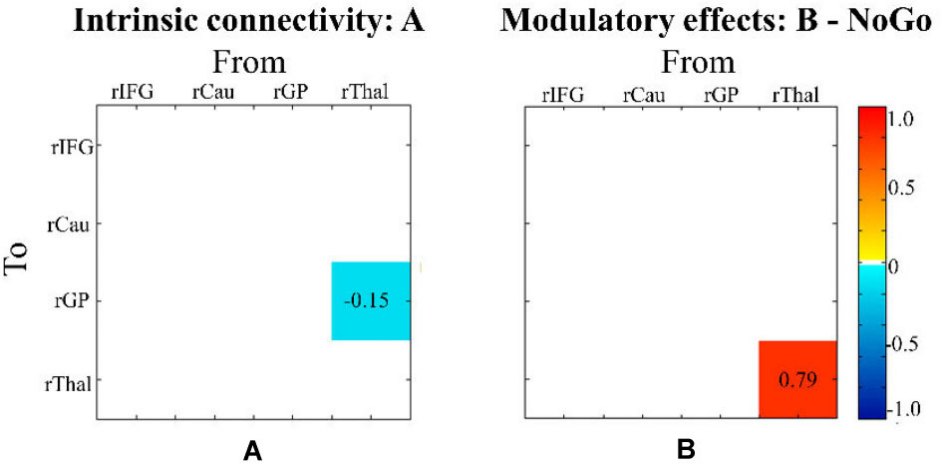
Sex effect on connectivity parameters in terms of A matrix and B matrix. (**A**) For intrinsic connectivity in A matrix, female participants showed a more negative influence from rThal to rGP compared to male participants. (**B**) In the NoGo condition, there is a greater self-inhibition in rThal in female than male participants in terms of B matrix. Effective connectivity strengths are displayed by the color ranging from yellow to dark red (i.e. excitatory connectivity) and from turquoise to dark blue (i.e. inhibitory). Parameters with stronger evidence (posterior probability > 95%) are presented.

Examining sex effects on intrinsic connectivity showed a negative influence from rThal to rGP in female compared to male participants across all experimental conditions (Fig. 3a). For the modulatory effects on connectivity, we found a greater self-inhibition in rThal in female than male participants in the NoGo condition (Fig. 3b). This suggests that for female participants, rThal exhibits reduced sensitivity to inputs from the other regions of the selected network during response inhibition.

### Brain Behavior Associations: Inhibitory Behavioral Performance and Connectivity Parameters

Examining associations between inhibitory performance on the behavioral level (NoGo performance) and connectivity parameters revealed a very strong evidence (posterior probability > 99%) that NoGo accuracy was positively associated with the directed connectivity from rThal to rIFG.

### DCM Analyses in the Left Hemisphere

**Figure 4:**
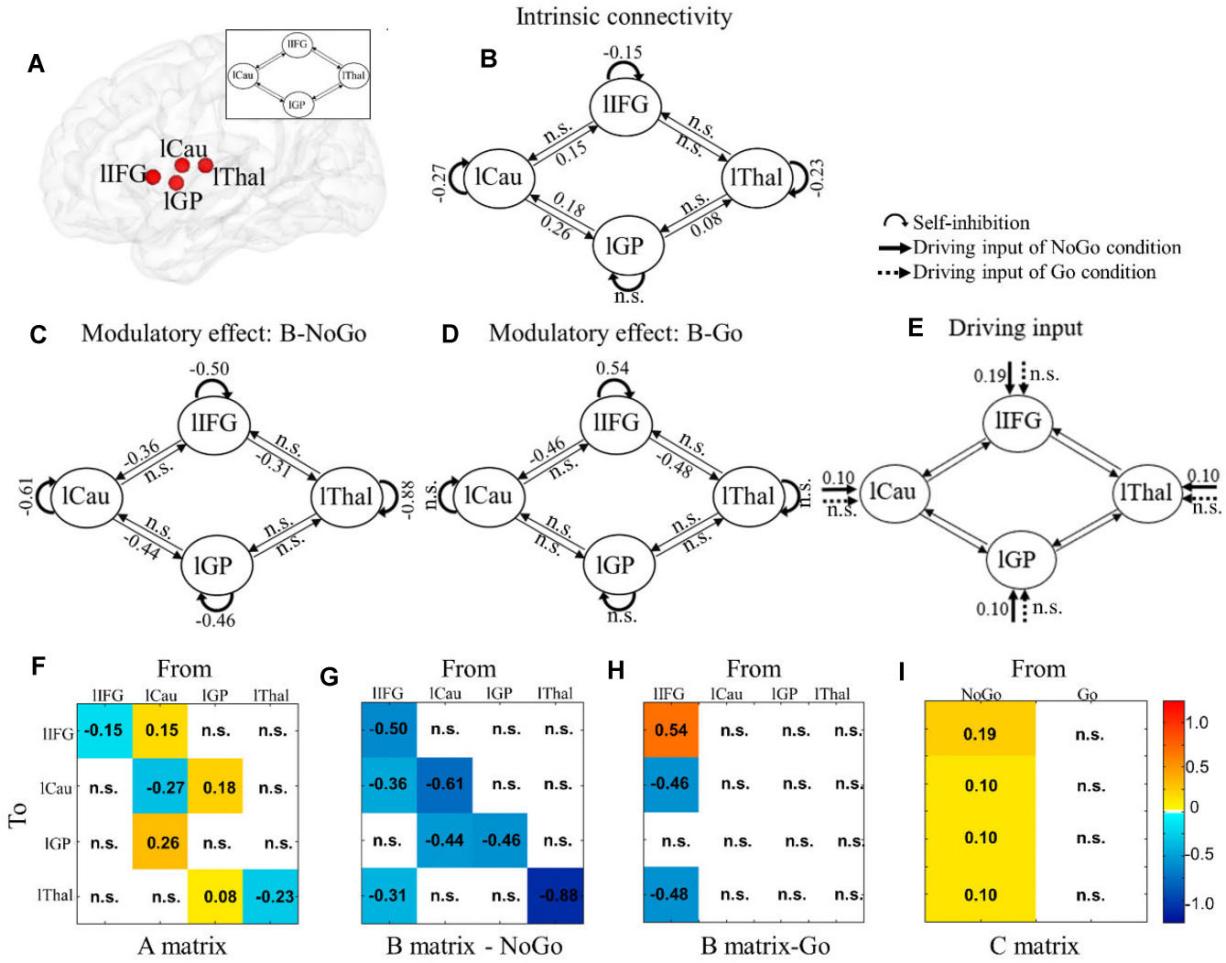
Location of regions included in the left model and group-level connectivity parameters. (**A**) Location of regions included in the left model. The A matrix: intrinsic connectivity independent of experimental conditions (**B, F**). The B matrix: modulatory effect on effective connectivity between regions and self-connections in the NoGo (**C, G**) and Go condition (**D, H**). The C matrix: driving inputs into ROI of NoGo and Go conditions (**E, I**). Values in matrices reflect the connectivity parameters. Effective connectivity strengths are displayed by the color ranging from yellow to dark red (i.e. excitatory connectivity) and from turquoise to dark blue (i.e. inhibitory). Parameters with stronger evidence (posterior probability > 95%) are presented and subthreshold parameters marked with “n.s.”.

To further validate the hemispheric asymmetry of the inhibitory control network, an identical model for the left hemisphere including lIFG, lCau, lGP, and lThal was tested (Fig. 4a). Participants with <10% explained variance were excluded and finally 82 participants (40 males, age: mean ± SEM = 21.24 ± 0.27 years) were included for the final DCM analyses. In contrast to the right model, no directed influences from IFG to subcortical regions were observed in terms of matrix A in the left model (Fig. 4b,f). Although the results showed modulatory effects of NoGo and Go conditions on the connectivity from IFG to Cau and Thal in both left and right models, the modulation effect of experimental condition on GP to Cau connectivity was only found in the right model (Fig. 4c,d,g,h). Additionally, the NoGo condition showed an inhibitory modulatory effect on the connectivity from Cau to GP in the left but not the right model and the Go condition showed an excitatory modulatory effect on the connectivity from Thal to IFG in the right but not the left one. Moreover, the two models had a similar pattern for the driving inputs of the NoGo condition on regions but not the Go condition (Fig. 4e,i). The different causal structure in the left and right model indicated a hemispheric asymmetry in the inhibition network. Additional Bayesian analyses confirmed the lack of a robust cortical-subcortical pathway in the left hemisphere (Supplementary Materials).

## Discussion

We capitalized on a combination of recent progress in biologically plausible causal hierarchical modeling (DCM-PEB) and a comparably large fMRI response inhibition dataset to determine causal information flow and key nodes within the extensively described basal ganglia-thalamocortical response inhibition circuits (Alexander *et al*., 1986, 1991; Alexander and Crutcher, 1990; Aron *et al*., 2007; Jahfari *et al*., 2019; Morein-Zamir and Robbins, 2015; Pfeifer *et al*., 2022; Schall and Godlove, 2012; Stuphorn, 2015; Verbruggen and Logan, 2009; Wei and Wang, 2016). Our neurocomputational model successfully validated a right-lateralized inhibitory control causal circuit and the best model showed significant intrinsic connectivity within this functional loop and captured an increasing causal influence of the cortical rIFG node on both the rCau and rThal as well as from the rGP to the rCau during inhibition. Direct comparison between different experimental conditions (e.g. NoGo and Go) revealed enhanced input into rIFG in terms of matrix C and increased connectivity from rIFG to rCau and rThal in the NoGo compared to the Go condition in terms of matrix B, suggesting a higher engagement of causal top-down cortical-to-subcortical control via the rIFG during inhibitory control. Although no sex differences were observed in inhibitory performance or BOLD activation, females exhibited decreased intrinsic connectivity from rThal to rGP and increased self-inhibition in rThal during the NoGo condition as compared to males. This indicates that a similar behavioral performance in response inhibition might be mediated by different brain processes in men and women, particularly in thalamic loops. Moreover, a higher NoGo response accuracy was associated with stronger causal information flow from the rThal to rIFG in the NoGo condition, suggesting a particular behavioral inhibitory relevance of this pathway. Finally, our findings showed different left and right model structures, suggesting a hemispheric asymmetry in the inhibitory control network and confirming a critical role of the rIFG in implementing response inhibition. Together, these findings identified a pivotal role of the rIFG and its effective connectivity with the rCau/rThal within the basal ganglia-thalamocortical circuit during response inhibition. Given that response inhibition deficits have been observed across a wide range of mental and neurological disorders, such findings may allow a more precise determination of target regions and circuits for neuromodulation strategies and personalized intervention.

Previous studies have underscored the predictive validity of the DCM approach based on hemodynamic responses changes (Bernal-Casas *et al*., 2017). A study by Bernal-Casas *et al*. combined optogenetic fMRI with DCM to examine cell-type-specific causal pathways among regions within the basal ganglia-thalamocortical network and found that effective connectivity pathways during D1- and D2-receptor-expressing medium spiny neuron stimulation significantly differed (Bernal-Casas *et al*., 2017). Furthermore, the DCM approach has also been validated based on electrophysiological time series with respect to estimating activity on the synaptic or neuronal level in both animal models (Moran *et al*., 2011; Papadopoulou *et al*., 2017; Rosch *et al*., 2018) and clinical studies in humans (Papadopoulou *et al*., 2015).

In the current study, causal modeling successfully determined a right lateralized inhibitory control causal circuit encompassing the rIFG, rCau, rGP, and rThal (Aron *et al*., 2003; Chevrier *et al*., 2007; Hung *et al*., 2018; Jahfari *et al*., 2011; Thompson *et al*., 2021). In terms of the matrix A, a significant rIFG-rCau-rGP-rThal loop was observed with rIFG exhibiting a negative influence onto rThal, alongside a positive information flow from from rThal to rGP and rCau to rIFG in the forward direction. In the backward direction, we found significant negative connectivity from rIFG to rCau and positive connectivity from rCau to rGP as well as rGP to rThal. A more lenient threshold additionally revealed rThal to rIFG connections (posterior probability of 57%). Importantly, accounting for behavioral task context revealed a significant positive modulatory effect on rIFG in both NoGo and Go condition in terms of matrix C, which was considerably stronger during response inhibition. The direct driving inputs into the rIFG are in line with its role in top-down target detection and attentional control in the context of response inhibition (Hampshire *et al*., 2010; Krämer *et al*., 2013) and indicate that the rIFG represents the key regulator of other nodes. Response inhibition impairments have been observed in several disorders and identification of the rIFG as critical input and top-down regulator for response inhibition opens new targets for regional or connectivity-based neuromodulation such as real-time neurofeedback, which has been established for these regions (Li *et al*., 2019; Weiss *et al*., 2022; Zhao *et al*., 2019). For instance, rIFG and response inhibition deficits have been determined in ADHD (Clark *et al*., 2007; Morein‐Zamir *et al*., 2014) and targeting the rIFG in ADHD may be a promising treatment.

In line with our hypothesis, the best model in terms of matrix B revealed strong evidence for causal effective connectivity from the rIFG to both rCau and rThal during response inhibition (posterior probability > 95%). This inhibitory pathway is consistent with previous reports on negative coupling between the rIFG and striatal regions during behavior control (Behan *et al*., 2015; Diekhof and Gruber, 2010). Notably, direct comparison using Bayesian contrast revealed a very strong evidence (posterior probability >99%) for increased modulatory connectivity from rIFG to rCau and rThal in the NoGo condition compared to the Go condition, suggesting the rIFG's driven engagement of cortical-to-subcortical top-down control during response inhibition. Previous animal models and human neuroimaging meta-analyses have consistently identified the rIFG as a key region implicated in dopaminergic and noradrenergic modulated inhibitory regulation (Bari *et al*., 2011; Hauber, 2010; Ott and Nieder, 2019; Pfeifer *et al*., 2022; Terra *et al*., 2020; Vijayraghavan *et al*., 2016; Zhukovsky *et al*., 2022), in particular during motor control and inhibition (Aron *et al*., 2003; Chamberlain and Sahakian, 2007; Puiu *et al*., 2020; Xu *et al*., 2016). Furthermore, both fronto-striatal and fronto-thalamic projections have also been extensively involved in response inhibition (Ahissar and Oram, 2015; Bosch-Bouju *et al*., 2013; Marzinzik *et al*., 2008; Phillips *et al*., 2021; Schmitt *et al*., 2017; Sommer, 2003; Tanaka and Kunimatsu, 2011).

In addition to the cortical-subcortical pathways significant excitatory connectivity was observed from the rGP to rCau during the Go condition and switched to inhibitory connectivity when response inhibition was required during the NoGo condition. Direct comparison confirmed a considerably stronger inhibitory influence of the rGP on the rCau during response inhibition (posterior probability >99%), suggesting that communication between basal ganglia nodes is crucial for context-appropriate behavioral response control. The involvement of this pathway is in line with extensive neurophysiological evidence showing that GABA inhibitory projections from the external segment of the GP to the striatum play an essential role in cancelling a planned response when it is inappropriate (Mallet *et al*., 2016; Wei and Wang, 2016) (but see also subthalamic nucleus to substantia nigra pars reticulata pathways in Hikosaka *et al*., 2006; Mallet *et al*., 2016). In addition, while numerous previous studies consistently demonstrated a right-lateralized fronto-striatal response inhibition circuit (Aron *et al*., 2003; Chevrier *et al*., 2007; Garavan *et al*., 1999; Hung *et al*., 2018; Jahfari *et al*., 2011), the present study additionally observed an inhibitory modulation effect of the NoGo condition on the effective connectivity between the left Cau to GP, suggesting that a left lateralized basal ganglia pathway may play an important role in action restraint.

With respect to sex difference analyses, we observed that females exhibited a lower intrinsic connectivity from rThal to rGP compared to male participants in the absence of performance differences, suggesting a different baseline basal ganglia-thalamic connectivity pattern independent of experimental contexts between males and females. In addition, we also found an increased modulatory effect of the NoGo condition on self-inhibition in the rThal in female, which indicates that female participants exhibited a reduced thalamic connectivity with other regions among the inhibitory control network compared to male participants. Given that previous studies reported an important role of the thalamus in relaying information and monitoring performance via reciprocal connections with the basal ganglia and PFC (Guillery, 1995; Phillips *et al*., 2021; Xiao *et al*., 2009; Tanaka and Kunimatsu, 2011), our findings may reflect a higher neural efficiency of this basal ganglia-thalamocortical circuit during response inhibition in females compared to males in the context of comparable performance in both groups. Moreover, while previous findings on sex differences in response inhibition performance and the underlying neural activity remained inconsistent (Chung *et al*., 2020; Gaillard *et al*., 2020, 2021; Li *et al*., 2006; Ribeiro *et al*., 2021; Sjoberg and Cole, 2018), similar findings have been reported in a previous study using a Go/NoGo task. This study reported significant sex differences on the neural response level in terms of functional connectivity in the absence of behavioral performance differences (Chung *et al*., 2020). However, it also has to be acknowledged that the findings by Chung *et al*., differ in important aspects from our findings, such as those authors observed greater functional connectivity between subcortical regions including thalamus and amygdala with other regions in females as compared to males. This may reflect the influence of age-related factors (the previous study was conducted in adolescents), given that males and females exhibit different neuromaturation of the inhibitory control circuits (Weafer, 2020). In addition, although the present findings suggest that our model was sensitive to biological variables and that separable information processes may underly response inhibition in men and women (see also Chung *et al*., 2020; Li *et al*., 2006), further research is needed to firmly verify the pivotal role of rIFG and its top-down control to subcortical rCau and rThal regions in response inhibition in the context of individual differences. Moreover, the functional relevance of the identified pathways was further underscored by a significant association between response inhibition performance and the causal influence from the rThal to rIFG in the NoGo condition, which demonstrates that this pathway involved in motor inhibition critically mediates behavioral success during inhibition (Wei and Wang, 2016).

Finally, our modeling tests confirmed a hemispheric asymmetry and support the critical role of right IFG circuit in response inhibition (Hung *et al*., 2018; Jahfari *et al*., 2011; Maizey *et al*., 2020). The different causal structures suggest a strong cortical-subcortical intrinsic connectivity and rIFG control on the right side, although the left model revealed a different causal structure and null hypothesis tests showed moderate evidence for the difference between NoGo and Go condition's modulatory effects on effective connectivity from lIFG to lCau and to rThal (e.g. lIFG to lCau: Bayes factor = 5.47; lIFG to lThal: Bayes factor = 8.20).

There are several limitations in the current study. First, in line with our main aim we did not account for emotional valence in the DCM model, which may affect response inhibition (Schimmack and Derryberry, 2005). Second, we focused on specific nodes that were based on established basal ganglia-thalamocortical circuits proposed by Alexander (Alexander et al., 1986, 1991; Alexander and Crutcher, 1990) (see also neuroimaging meta-analysis: Hung *et al*., 2018). Other regions such as the STN (Aron *et al*., 2016; Aron and Poldrack, 2006; Chen *et al*., 2020) could be integrated in future studies. Third, although DCM has advantages in testing directed connectivity and causal pathways between regions, it also has a number of limitations. For instance, the approach uses a Bayesian information procedure and as such is stringently dependent on the priors (Friston *et al*., 2003). Moreover, the approach assumes that activity in the neurons forming an assembly is conform which does not adhere to the actual physiological properties (Friston *et al*., 2003).

## Conclusions

In conclusion, our findings demonstrated a critical role of the rIFG as well as top-down cortical-subcortical control from the rIFG to rCau and rThal in response inhibition. The nodes and pathways of the model were sensitive to biological and performance variations. The nodes and pathways may represent promising targets to improve response inhibition in mental disorders.

## Supplementary Material

kkad016_Supplemental_File
